# Towards the integration of mental practice in rehabilitation programs. A critical review

**DOI:** 10.3389/fnhum.2013.00576

**Published:** 2013-09-19

**Authors:** Francine Malouin, Philip L. Jackson, Carol L. Richards

**Affiliations:** ^1^Département de Réadaptation, Faculté de Médecine, Université LavalQuébec, QC, Canada; ^2^Centre Interdisciplinaire de Recherche en Réadaptation et Intégration SocialeQuébec, QC, Canada; ^3^École de Psychologie, Université LavalQuébec, QC, Canada; ^4^Centre de Recherche de l'Institut Universitaire en Santé Mentale de QuébecQuébec, QC, Canada

**Keywords:** motor imagery, motor imagery training, mental practice, stroke rehabilitation, motor skill learning, stroke, Parkinson's disease, neurological rehabilitation

## Abstract

Many clinical studies have investigated the use of mental practice (MP) through motor imagery (MI) to enhance functional recovery of patients with diverse physical disabilities. Although beneficial effects have been generally reported for training motor functions in persons with chronic stroke (e.g., reaching, writing, walking), attempts to integrate MP within rehabilitation programs have been met with mitigated results. These findings have stirred further questioning about the value of MP in neurological rehabilitation. In fact, despite abundant systematic reviews, which customarily focused on the methodological merits of selected studies, several questions about factors underlying observed effects remain to be addressed. This review discusses these issues in an attempt to identify factors likely to hamper the integration of MP within rehabilitation programs. First, the rationale underlying the use of MP for training motor function is briefly reviewed. Second, three modes of MI delivery are proposed based on the analysis of the research protocols from 27 studies in persons with stroke and Parkinson's disease. Third, for each mode of MI delivery, a general description of MI training is provided. Fourth, the review discusses factors influencing MI training outcomes such as: the adherence to MI training, the amount of training and the interaction between physical and mental rehearsal; the use of relaxation, the selection of reliable, valid and sensitive outcome measures, the heterogeneity of the patient groups, the selection of patients and the mental rehearsal procedures. To conclude, the review proposes a framework for integrating MP in rehabilitation programs and suggests research targets for steering the implementation of MP in the early stages of the rehabilitation process. The challenge has now shifted towards the demonstration that MI training can enhance the effects of regular therapy in persons with subacute stroke during the period of spontaneous recovery.

## Introduction

The ever-increasing number of publications attests to clinician expectations of mental practice (MP) through motor imagery (MI) as a means of promoting the recovery of motor function (for a review see Malouin and Richards, 2013). MP not only provides a unique opportunity to increase the number of repetitions in a safe and autonomous manner without undue physical fatigue, but it also allows the mental rehearsal of motor tasks when and where the patient wants to, or is able to, practice. Furthermore, MP enables the rehearsal of more demanding or complex motor tasks (e.g., walking, writing) when physical practice is impossible or too difficult. Yet, despite these obvious advantages, MP is a complex mental process that is not readily amenable to be integrated into clinical practice. To date, in most published studies, MP has been used within constrained research environments to meet the requirements associated with research methodology.

As highlighted by several review papers concerning the use of MP in rehabilitation, (van Leeuwen and Inglis, 1998; Jackson et al., 2001; Braun et al., 2006; Dickstein and Deutsch, 2007; Zimmermann-Schlatter et al., 2008; Dijkerman et al., 2010; Malouin and Richards, 2010, 2013) there are marked differences in designs, research protocols, training regimens and outcome measures among the growing number of studies. Despite this heterogeneity, positive effects of MP on motor function have been generally reported. However, Braun et al. (2006), in a systematic review of five selected randomized controlled trials (RCT), stated that although there was some evidence that MP as an adjunct therapeutic intervention had beneficial effects on arm function, they were not able to draw definite conclusions and stated that further research with a clear definition of the content of the MP and standardized outcome measures were needed. In a more recent review that included six studies, Barclay-Goddard et al. (2011) also concluded that the combination of MP with other treatments appeared to be more effective than other treatments alone to improve upper extremity function. Based on their assessment with the PEDro scale, the quality of the evidence was moderate. Likewise, in their systematic review of 15 studies, Nilsen et al. (2010) attested that when MP was added to physical practice (PP), it was an effective intervention. Nevertheless, they also mentioned that further research was needed to identify those patients most likely to benefit from training, the optimal dose, and the most effective protocols.

These reviews, however, did not include the findings originating from recent multicenter RCTs (Bovend'Eerdt et al., 2010; Ietswaart et al., 2011; Braun et al., 2012; Timmermans et al., 2013) in subacute patients that have attempted to integrate MI training in regular rehabilitation programs. Not only did the addition of MP to conventional training on all tasks fail to yield better functional outcomes than conventional training, but the low compliance of therapists (Bovend'Eerdt et al., 2010; Braun et al., 2010, 2012) and realities related to patients such as advanced age of those in nursing homes (Braun et al., 2012) point to some of the difficulties encountered when attempting to introduce MP into regular clinical practice. The findings of two recent RCTs, (Ietswaart et al., 2011; Timmermans et al., 2013) did not confirm the additional benefits of including MI training in the rehabilitation program aimed at improving upper limb function. Despite meticulously designed MI training that included a variety of approaches (action observation through mirror therapy, implicit imagery, and self-practice), patients with subacute stroke did not show additional gains in the performance of activities of daily living (ADL) (Ietswaart et al., 2011). Altogether, these latest findings reflect the complexity of integrating MP into regular rehabilitation programs. Thus, this review scrutinizes the current application of MP, and from this analysis proposes a framework for its integration into usual rehabilitation programs.

## Rationale underlying MI training

With the turn of the twenty-first century, we have witnessed the emergence of clinical studies designed to investigate the effects of MP on the relearning of motor skills in persons with stroke. The rationale for using MI training to promote the relearning of motor function arises from research on the functional correlates that MI shares with the execution of physical movements. It is now widely recognized that the duration of mentally simulated actions usually correlates with the duration of real movements (temporal coupling), that the simulation of movements evokes similar autonomic responses and that the imagination of an action or its physical execution engage largely similar neural networks (Decety and Boisson, 1990; Decety et al., 1991; Decety and Jeannerod, 1995; Wuyam et al., 1995; Decety, 1996; Decety and Grèzes, 1999; Lafleur et al., 2002; Malouin et al., 2003; Fusi et al., 2005; Munzert and Zentgraf, 2009; Hétu et al., 2013). These similarities led to the notion of functional equivalence. Thus, real and covert movements during MI obey similar principles and share similar neural mechanisms, likely explaining the beneficial effects of MP on motor performance (Jeannerod, 1995).

### MI training (MP) in healthy individuals: skill learning

Much of the evidence for using MI in the training of motor function is based on findings from studies that examined the effect of MI training in healthy adults (Yue and Cole, 1992; Pascual-Leone et al., 1995; Jackson et al., 2003; Allami et al., 2008; Olsson et al., 2008; Reiser et al., 2011). These studies have shown that MI training *alone* can significantly promote the learning of a novel motor skill, but it is important to keep in mind that such training needs to be very intensive. For instance, subjects who rehearsed mentally a sequence of foot movements for 5 days, demonstrated, significant improvement of their performance after 1500 mental repetitions (Jackson et al., 2003). Likewise, when learning a complex sequence of finger movements, subjects in another study practiced physically (PP) or mentally (MP) 2 h a day for 5 days to learn the task. After 5 days, while best results were found in the PP group, the MP group had significantly improved in comparison to a control group, indicating that MP was effective, but not as effective as PP (Pascual-Leone et al., 1995). However, after one 2-h physical training session, subjects in the MP group reached the same level of performance attained by those in the PP group who had 10 h of physical practice. Thus, although the learning of a motor skill requires hundreds of repetitions, the number of physical repetitions to obtain similar gains can be less if subjects rehearse mentally prior to PP, indicating that MI can exert priming effects on subsequent PP. Similar priming effects have been observed in a study wherein subjects had to learn a precision grasp task. While it took 240 physical repetitions to learn the task, subjects who first did 120 mental rehearsals needed only 120 physical repetitions to reach an equivalent performance (Allami et al., 2008). These examples hint at the potential use of the priming effects of MI training in rehabilitation. For instance, the findings of Pascual-Leone et al. (1995) suggest that if only MI is used in the early rehabilitation phase when PP is not possible, (e.g., walking), then when PP becomes possible, less PP will be required to attain a given level of motor performance. Whereas, findings from Allami et al. (2008) suggest that when PP is possible, combining MP and PP will require less PP to attain a similar level of motor performance. These findings in healthy individuals also illustrate how the addition of MP to a rehabilitation program, should not necessarily entail an increase in the overall burden of therapy, but could in some cases simply imply a trade-off from one form of therapy to another.

### Mental practice in sport

Applications of MI training to neurological rehabilitation are also guided by findings in athletes who use imagery to practice motor skills and enhance skill acquisition or to facilitate the actual performance of a learned skill, as well as for motivation, self-confidence and anxiety reduction (Feltz and Landers, 1983; Janssen and Sheikh, 1994; Murphy, 1994; Rushall and Lippman, 1998; Guillot and Collet, 2008; Munzert and Lorey, 2013). Studies have clearly shown that the largest gains in motor performance are obtained when MP is combined with PP, and that MP alone yields better results than no training at all (Richardson, 1967a,b; Ryan and Simons, 1982; Feltz and Landers, 1983; Hall et al., 1990, 1992, 1998; Driskell et al., 1994; Brouziyne and Molinaro, 2005; Weinberg, 2008). MP is also used alone, without concomitant physical practice. For instance, prior to a competition it is used to refresh kinesthetic memory, especially for complex routines (gymnastics) or part of routines that are quite demanding physically, or between physical training sessions to maintain performance level (Rodgers et al., 1991; Murphy, 1994; Rushall and Lippman, 1998). For performance preparation, the focus is on factors that enhance performance such as motivation or activation (Paivio, 1985; Rushall and Lippman, 1998). Athletes imagine their forthcoming performance in real time to “get a feeling” for how to respond to the requirements of a task (Munzert and Lorey, 2013). Overall, athletes seem to use motor imagery more in conjunction with competition than with practice, perhaps because of its very important motivational function (Hall et al., 1990; Munroe et al., 2000; Munzert and Lorey, 2013). Several models of MP in sports (for a review see Guillot and Collet, 2008) include both a cognitive (learning) and a motivational (emotion) function; besides potential motor priming effects, athletes who imagine themselves performing well may become more motivated to practice harder and to compete more intensely.

### Research protocols in neurological rehabilitation (Tables 1 and 2).

Given the large variety of research protocols that have been developed to examine the impact of MP, a classification of the types of protocols was made based on common characteristics. An important aspect that has surprisingly not often been systematically reviewed is whether MP is provided alone or in combination with physical practice. Next is the manner in which MP is provided: through audiotapes or guided by a therapist (one to one). It is also important to consider when MP is combined with PP, if it is within the same training session or a few hours apart in separate sessions. Thus, three modes of MI delivery have been proposed based on the analysis of the research protocols of 27 clinical studies in persons with stroke (*n* = 25) or with Parkinson's disease (*n* = 2) (Table 1). This classification is arbitrary, but reflects the reality of how MP is used in clinical practice. The first two modes (1 and 2) include protocols wherein MP and PP are combined, whereas, the third mode includes protocols with only MI training without specific physical training (mode 3). When mental and physical practice are combined, they are either carried out in separate sessions (mode 1: separate sessions) through different approaches (audiotapes: 1A or one to one: 1B) or provided in the same session (mode 2: concurrent session) under the guidance of a therapist with series of physical repetitions alternating with mental repetitions (Table 1). A first step in the analysis was to examine the type of tasks trained (ADL using the upper limbs or mobility and locomotor) across the three modes of MI delivery. Table 2, shows that 77% (21/27) combined physical and mental training (modes 1 and 2) and only six out of the 27 studies used MI alone (mode 3) and this allocation was similar whether training ADL (12/16: 75%) or mobility and gait (9/11: 82%). However, 56% of the MI studies of ADL tasks opted for audiotape delivery (mode 1A). This mode, however, was never used for training mobility and gait, instead, guided MI (one to one) was used in separate sessions (mode 1B: 36%) or with MP and PP provided in the same session (mode 2: 46%).

**Table 1:** **Modes of MI delivery and examples of tasks**.

**Research protocols**	**Tasks**
**(1) PP AND MI PROVIDED IN SEPARATE SESSIONS SEPARATE MODE OF MI DELIVERY**	
(1A) PP + MI (relaxation + audiotape)	ADL
(1B) PP + Guided MI (one to one)	Gait, ADL
**(2) PP AND MI PROVIDED IN THE SAME SESSION CONCURRENT MODE OF MI DELIVERY**	
Guided MI (one to one): ratio 1 PP:10 MP; 1PP:5MP	Rising-up from a chair/sitting down, reach/grasp; gait
**(3) MI ALONE: MI**	
Guided MI (one to one)	Gait, ADL, sequence of finger movements

**Table 2:** **Modes of MI delivery in the research protocols of the 27 clinical studies reviewed**.

**Research protocols**	**ADL**				**Research protocols**	**Mobility and gait**			
	***N***	**%**	**Chronic (*N*)**	**Non-chronic (*N*)**		***N***	**%**	**Chronic (*N*)**	**Non-chronic (*N*)**
Mode 1A	9	56	8	1	Mode 1A	0	0	0	0
Mode 1B	1	6	0	1	Mode 1B	4	36	4	0
Mode 2	2	13	0	2	Mode 2	5	46	5	0
Mode 3	4	25	1	3	Mode 3	2	18	2	0
Total	16	100	9	7	Total	11	100	11	0

Another observation is that 74% of the studies (20/27) included patients more than 6 months post-stroke (chronic phase) after they had completed formal rehabilitation (Table 2). At such a stage, motor improvement is unlikely to be associated with spontaneous neurological recovery and thus functional improvement can be more readily attributed to a given intervention. The remaining studies were carried out with patients in the subacute phase post-stroke and all targeted ADL training with the upper limbs (Crosbie et al., 2004; Müller et al., 2007; Bovend'Eerdt et al., 2010; Riccio et al., 2010; Ietswaart et al., 2011; Braun et al., 2012; Timmermans et al., 2013).

Because MP is an adjunct to PP, it is hypothesized that patients receiving MP in addition to PP will demonstrate larger gains compared to a control group receiving only PP. In most controlled studies, a placebo intervention equivalent in time to the MP is provided to the control group to make up for extra contact time. To control for attention, the placebo usually consists of mental activities unrelated to movement imagery and can be delivered on tape (relaxation exercises; information about stroke, puzzles, etc.) or by audiovisual means (video, computer program, pictures, TV programs, etc.) with a content unrelated to the tasks practiced. This control for contact-time is not always provided (Page et al., 2009; Riccio et al., 2010; Lee et al., 2011; Cho et al., 2013) or the sham intervention consists of additional PP (Braun et al., 2012; Timmermans et al., 2013).

## The three modes of MI delivery

### Separate mode of MI delivery (mode 1A and 1B)

In mode 1 (Table 1), the MP and PP are provided in separate sessions with MI training delivered either through audiotaped scripts (mode 1A) or guided by a therapist on a one to one basis (mode 1B). The MI training is provided later in the day (Page, 2000; Page et al., 2001, 2005, 2007, 2009, 2011; Riccio et al., 2010) or right after physical and/or occupational therapy training sessions (Yoo and Chung, 2006; Bovend'Eerdt et al., 2010; Hwang et al., 2010; Lee et al., 2011; Nilsen et al., 2012; Cho et al., 2013).

#### MI training of upper limb ADL tasks (Table 3: mode 1A)

In most studies involving upper limb ADL training, the MP was carried out in a quiet environment while patients lay supine or sat and listened to an audiotape describing the motor tasks to be rehearsed mentally (Page, 2000; Page et al., 2001, 2005, 2007, 2009, 2011; Yoo et al., 2001; Riccio et al., 2010; Nilsen et al., 2012). Largely influenced by cognitive and sport psychology (Suinn, 1984, 1985; Paivio, 1985; Sordoni et al., 2000; Cupal and Brewer, 2001), an audiotaped MI training session typically consists of a period of relaxation (3–5 min) wherein the patients are asked to imagine themselves in a warm and relaxing place and to contract and relax muscles. This is followed by 10–20 min of suggestions for internal, cognitive polysensory (visual and kinesthetic cues) images related to using the affected arm in one of several functional tasks. The tape concludes with 3–5 min of refocusing into the room (as described in Page et al., studies). Therefore, about 6–10 min of each session is not devoted to mental rehearsal as such.

Although the rationale for using audiotape delivery has never been explicitly justified, it is likely inspired by Paivio (1985), who underlined the importance of an accurate representation of the skill to be practiced, and thus proposed that language was an efficient way of activating imagery content. Likewise, the addition of relaxation prior to MI training was added to heighten concentration, promote vividness of MI, as well as to enhance performance and attention (Hall and Erffmeyer, 1983; Suinn, 1984, 1985; Sordoni et al., 2000; Cupal and Brewer, 2001).

**Figure 1:**
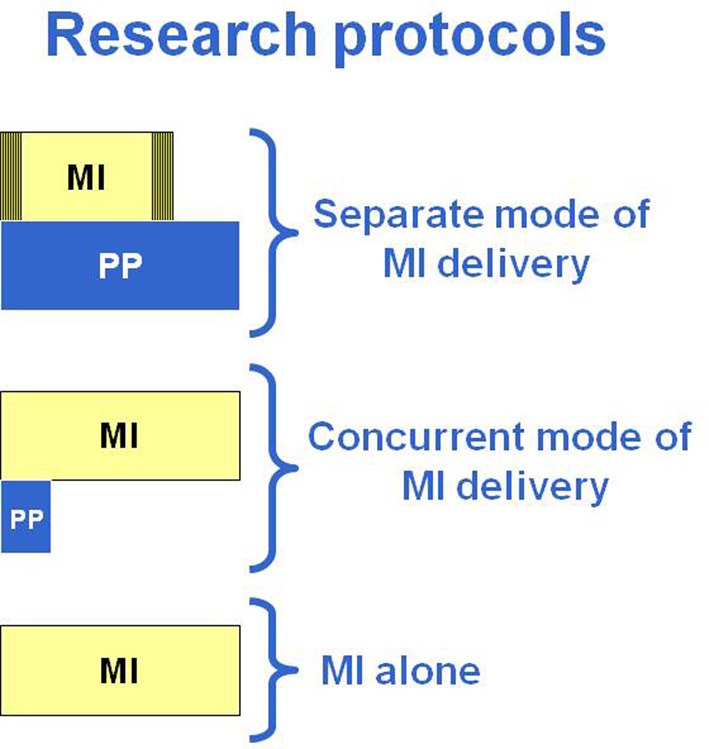
**Schematic representation of the time dedicated to Motor Imagery training (MI) and Physical Practice (PP) for each mode of MI delivery**. The vertical lines indicate the proportion of time for relaxation (prior to MI) and refocusing (after MI). More time is allotted to PP than MI training in the separate mode of MI delivery, whereas in the other two modes more time is devoted to MI training.

The time allotted to mental rehearsal itself can be as little as 5 out of 10 min (Page et al., 2001), 8 of 18 min (Nilsen et al., 2012), as much as 15–20 min of a 30 min session (Page et al., 2005, Page et al., 2007, 2009) or as much as 48 out of 60 min (Riccio et al., 2010). Table 3 gives a general idea of the total number of hours dedicated to MP and PP. Because MI training sometimes includes other components (e.g., relaxation, refocusing, implicit imagery) it was decided to determine the proportion of time dedicated to mental rehearsal alone. Thus, in the mental practice section (5th column in the Table), when there are two numbers the first number estimates the total number of hours allotted to mental rehearsal, whereas, the second number gives the total number of hours for the whole MI training session (including relaxation, etc.). When comparing the total hours of PP (column 4) and MP (column 5) sections in Table 3, it is clear that more time is devoted to physical than mental practice; the proportions are schematized in Figure 1. Although these numbers provide a general estimate arrived at from the descriptions found in the methods of the published articles, they illustrate the large variability in MI training regimens across studies. In addition, these numbers do not include self-practice or unsupervised training activities, since generally no information was provided about the compliance. About 3–6 tasks are rehearsed mentally over the training period, except in one study that included as many as 12 tasks (Riccio et al., 2010).

**Table 3:** **Characteristics of MI training studies for upper limb tasks**.

**^Res Prot^/Study**	***N***	**TSS**	**PP**	**Mental practice**				**Mean change scores**				
			**Hrs**	**Hrs**	**Eval**	**✓**	**Tasks**	**ARAT**	**FMA**	**Jebsen(%)**	**AFT**	**Midx**
^1A^Page, 2000	E:8	1.8 years	6	2–4	NO	NO	?	NA	7.8	NA	NA	NA
	C:8		6						4.7			
^1A^Yoo et al., 2001	E:3	2, 12–16 months	0	2	NO	NO	1	NA	NA	NA	NA	NA
^1A^Page et al., 2001	E:8	6–11 months	18	1.8–3	YES	NO	3	16.4	13.8	NA	NA	NA
	C:5		18					−1.4	2.9			
^1A^Page et al., 2005	E:6	2 years	6	4–6	NO	NO	3	10.7	NA	NA	NA	NA
	C:5		6					4.6				
^1A^Page et al., 2007	E:16	38 months	6	4–6	NO	NO	3	7.8	6.7	NA	NA	NA
	C:16	45 months	6					0.4	1.0			
^1A^Page et al., 2009	E:5	13–45 months	^*^15	10–15	NO	NO	5	15.4	7.8	NA	NA	NA
	C:5		^*^15	No sham				8.4	4.1			
^1A^Riccio et al., 2010	E:18	2 months	45	12–15	NO	NO	12	NA	NA	NA	14.1	11.4
NC	C:18			No sham							0.83	1.9
^1A^Page et al., 2011	E:8		15	5–10	NO	NO	5	2.8	2.7			
	E:6	36 months	15	15–20			5	1.7	4.0	NA	NA	NA
	E:7		15	25–30			5	1.8	5.3			
	C:8		15					0.2	2.2			
^1A^Nilsen et al., 2012	E:5	43 months	6	1.6–4	YES	YES	3	NA	9.6	33		
	E:6	20 months	6	1.6–4					10.6	42	NA	NA
	C:6	33 months	6						3.8	−13		
^1B^Bovend'Eerdt et al., 2010	E:15	22 weeks	Usual therapy	6.5	NO	NO	ALL	4.8	NA	NA	NA	NA
NC	C:15	16 weeks						4.3				
^2^Crosbie et al., 2004	E:14	10–42 days	^E^28 rep	^E^280 rep	NO	YES	1	NA	NA	NA	NA	^E^41
NC												10–60
^2^Braun et al., 2012	E:18	4–6 weeks	Usual therapy	?	NO	NO	ALL	NA	NA	NA	NA	17
NC	C:18											21
^3^Stevens and Stoykov, 2003	E:1	1, 2 years	0	12	NO	NO	2	NA	10	67	NA	NA
	E:1		0	12					12	33		
^3^Müller et al., 2007	E:6	1 month	0	10	NO	YES	1	NA	NA	30	NA	NA
NC	E:6		10	0						40		
	C:5		0	0						0		
^3^Ietswaart et al., 2011	E:39	82 days	Usual therapy	8–9	YES	NO	12–14	5.9	NA	NA	NA	NA
NC	C:31							5.3				
	C:31							7.3				
^3^Timmermans et al., 2013	E:18	1 month	Usual therapy	15	YES	NO	6	NA	4.0	NA	NA	NA
NC	C:14								5.0			

PP, physical practice; TSS, mean time since stroke; Hrs, hours; Eval, MI assessment; ✓, manipulation checks; Tasks, number of tasks rehearsed mentally; ARAT, Arm Research Assessment Test (Max: 57); FMA, Fugl-Meyer Assessment (motor upper extremity: max 66); AFT, Arm functional test (timed test); Jebsen, timed test; Midx, Motricity Index (Max:100);

Res Prot 1A, 1B, 2, 3 , research protocols described in Table 1; NC, non-chronic strokes;

E Estimated from text and figures;

* 45 h with a glove on sound hand: constraint induced therapy (CIT); No sham, no control for contact time; NA, not applicable; ?, unspecified.

As for the physical practice part, based on descriptions found in the published protocols, the physical training generally focused on ADL tasks rehearsed later in separate MI training sessions (Page et al., 2005, 2007, 2009, 2011; Nilsen et al., 2012). Patients in these studies who were at a chronic stage with stable motor functions engaged in physical training sessions that ranged from 30 to 60 min, 2–5 times a week, over 2–10 weeks for a total duration ranging from 6 to 45 h (median of 9 h). Training generally involved 3–5 tasks (Table 3: mode 1A).

#### Gait tasks (Table 4: mode 1B)

Although MI training of gait was also provided in a separate session after PP, it was not delivered through audiotaped scripts, but guided on a one to one basis by a therapist (Hwang et al., 2010; Lee et al., 2011; Deutsch et al., 2012; Cho et al., 2013). However, relaxation prior to MI training was also used before MI training (Hwang et al., 2010) or after (Cho et al., 2013). In the four studies on gait training (Yoo and Chung, 2006; Hwang et al., 2010; Lee et al., 2011; Cho et al., 2013), MI training followed physical training and technical support was also used to illustrate what should be imagined; for instance, patients watched videos to learn about walking or to identify their own gait problems (Hwang et al., 2010; Lee et al., 2011).

As for the physical training component, it consisted of treadmill training (Lee et al., 2011; Cho et al., 2013) for 30 min, 3 times a week for 6 weeks (total: 9 h). In another study (Hwang et al., 2010), 1 h of regular physical therapy was provided 5 days a week for 4 weeks (total: 20 h), but the amount of time dedicated to gait was not specified. These examples illustrate the marked variations in intensity of physical training (20 h over 4 weeks: 5 h/week, vs. 9 h over 6 weeks: 1.5 h/week) across studies. Thus, given such variation in both the intensity and the specificity of the physical training, the assumption is that the contribution of PP to the overall effects is also variable.

#### Multiple Tasks (Table 3: mode 1B)

Recently, attempts have been made to integrate MI training into usual clinical rehabilitation programs without increasing the total time of therapy (Bovend'Eerdt et al., 2010). MI training was used in the training of multiple tasks (upper and lower limbs, locomotion, etc.) instead of concentrating on a few selected ADL or mobility tasks. However, given the compliance problems with the therapists, the total amount of MI training could only be estimated by the authors (about 6.5 h) and the amount of physical practice was not reported.

### Concurrent mode of MI delivery (mode 2)

In other studies, mental and physical repetitions were provided in the same training session with series of physical repetitions alternating with the mental repetitions. The ratio of MP to PP is variable across studies, with the number of MP increasing progressively from 2MP:1PP up to 10MP: 1PP (Crosbie et al., 2004; Malouin et al., 2004a, 2009; Tamir et al., 2007; Deutsch et al., 2012).

The rationale behind this approach is to tap into the priming effects of MI on subsequent physical performance (Pascual-Leone et al., 1995) and to decrease the number of physical repetitions (Allami et al., 2008; Reiser et al., 2011). In addition, visual and kinesthetic information acquired during each physical repetition refreshes the movement memory of the motor task and assists in the accuracy and vividness of the mental images and sensations for the next series of mental repetitions (Crosbie et al., 2004; Malouin et al., 2004a,b, 2009). It was also found that the timing (functional equivalence) of the motor task being rehearsed mentally improved when mental repetitions alternated with physical repetitions, thus suggesting that the afferent information is helpful for consistent reproduction of the next imagined movement (Courtine et al., 2004). Since MI training with this mode of delivery involves a large number of repetitions (up to 100 mental repetitions for 10 physical repetitions/session), most studies focus on one task at a time (e.g., reaching for a cup, standing up) and progression is made by increasing the difficulty of the task (e.g., biomechanical constraints) in steps tailored to individual requirements. Moreover, with the latter approach the total number of repetitions rather than the duration of the sessions is the key factor. Overall, more mental than physical repetitions are provided (Figure 1) and several hundred repetitions are targeted (Crosbie et al., 2004; Malouin et al., 2009), to promote motor learning (Nudo et al., 1996) and enhance the effects of physical practice (Allami et al., 2008; Reiser et al., 2011). For example, when combined with about 1100 mental repetitions, improved motor performance of the sit-to-stand task was obtained (Malouin et al., 2009) with only 100 physical repetitions, well below the 450–600 physical repetitions needed to promote motor learning of the sit-to-stand task (Monger et al., 2002; Barreca et al., 2004).

#### Upper limb ADL tasks (Table 3: mode 2)

Only two studies combined MP and PP within the same session for the training of upper limb ADL tasks (Crosbie et al., 2004; Braun et al., 2012). In one study (Crosbie et al., 2004) that focused on the training of reaching and grasping movements, the proportion of MP to PP was 10MP: 1PP and overall the number of mental repetitions was estimated to be 280 (for 28 physical repetitions).

#### Gait and mobility tasks (Table 4: mode 2)

In studies using mental and physical repetitions for training mobility tasks in the same session (e.g., rising from a chair and sitting down), the ratio of MP to PP for training varied: 5MP: 1PP (Malouin et al., 2004a), 10MP: 1PP (Malouin et al., 2009) and 3MP: 3PP (Tamir et al., 2007). The total repetitions, over 12 training sessions with a ratio of 10MP: 1PP could be as much as 1100 mental repetitions and 100 physical repetitions (about 10 h of contact with the patient). In a recent case study (Deutsch et al., 2012), a ratio of 5MP: 1PP was used for gait training.

#### Multiple tasks (Table 3: mode 2)

The only study that used MI training for multiple tasks was carried out in a nursing home in patients with subacute stroke (Braun et al., 2012). Several tasks involving both the upper and lower extremities were trained. Given the problems with compliance, no information about the amount of training was reported.

### MI alone (mode 3)

While modes 1 and 2 of MI delivery also provide physical practice of the tasks rehearsed mentally, in mode 3, physical practice specific to the tasks rehearsed mentally was not included, and MI training was provided on a one to one basis. The rationale for using MI alone, as underlined in one study, was the need to show the benefit of MP alone to confirm its role in brain plasticity (Ietswaart et al., 2011). In reality, it is difficult to completely remove all physical training, especially in patients with subacute stroke who are engaged in usual rehabilitation programs (Müller et al., 2007; Ietswaart et al., 2011; Timmermans et al., 2013) or when MI training of gait is carried out in ambulatory patients (Dunsky et al., 2008) since one expects that the patients continue to be engaged in daily activities. However, one can assume that compared to the other studies that included intensive physical training specific to the tasks trained mentally, the amount of physical practice was likely much less.

#### Upper limb ADL tasks (Table 3: mode 3)

Some studies using MP alone focused on the learning of a finger movement sequence (Müller et al., 2007), wrist movements (Stevens and Stoykov, 2003), or upper limb ADL tasks (Ietswaart et al., 2011; Timmermans et al., 2013). In these studies MI training was delivered under the guidance of a therapist or with a computer interface (Stevens and Stoykov, 2003) and the total amount of MI training was 10 (Müller et al., 2007) and 12 h (Stevens and Stoykov, 2003), respectively. Training generally involved 1–2 tasks, but in two recent studies (Ietswaart et al., 2011; Timmermans et al., 2013) carried out in the subacute phase, several tasks (6–14) were rehearsed mentally with training carried out on a one to one basis with the addition of an audiovisual (DVD) interface (Timmermans et al., 2013). In one study, MI training included mirror therapy and implicit motor imagery (Ietswaart et al., 2011).

#### Gait and mobility tasks (Table 4: mode 3)

MP alone for gait training was used by Dunsky et al. (2008). Guided by a therapist, the training included a relaxation period (2–3 min) followed by mental rehearsal of walking (10 min) and ended with a refocusing period (2 min). The MI training for the sit-to-stand task (Guttman et al., 2012) used a similar protocol, starting with relaxation and ending with a refocusing section.

In conclusion, the large diversity of protocols used to date reflects the search for an optimal approach. The rationale underlying the selection of a given protocol or training regimen is not always clearly defined. While the influence of former studies is at times clearly expressed, there is usually no justification for the selection of the intervention parameters.

## Factors influencing MI training outcomes

### Adherence to MI training

**Figure 2:**
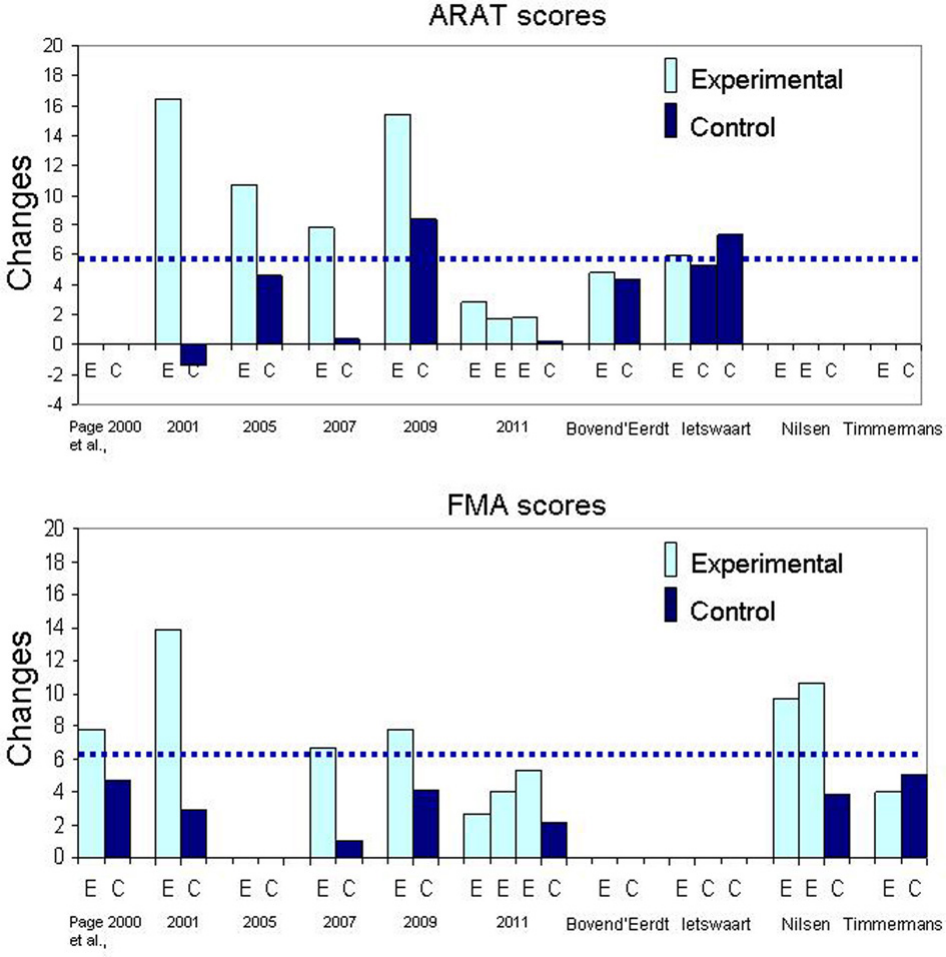
**Bar graphs illustrating the mean changes in Arm Research Action Test (ARAT) and Fugl-Meyer Assessment (FMA)**. The dotted line represents the Minimal Clinically Important Difference (MCID) for each outcome measure.

As reviewed above, there is much variability in the content of MI training and the time dedicated to mental rehearsal. Moreover, for most studies, it is impossible to estimate the number of mental repetitions over the training period (dose) since these are rarely counted. There is not only much variability in both the amount of time dedicated to MI training and in the mode of MI delivery, but one must also ponder whether the mental rehearsal was done correctly. Very few studies assessed the MI ability of participants (Tables 3, 4), so there is no certainty that the patients were able to engage in motor imagery at the start of the study. In addition, even if they were good imagers, without manipulation checks to control whether they conformed to the instructions, it is impossible to confirm their adherence to the MI training. The findings that larger doses of MI training (Figure 2; comparison of 20, 40, and 60 min per session) delivered through audiotapes (Page et al., 2011) yielded inconsistent and not clinically meaningful results (very small ARAT gains and low trends of dose-related FMA gains) raise the question of patient adherence to instructions for the longer durations (e.g., mental fatigue, boredom) and further emphasize the need to control for patient compliance during MI training. Monitoring for compliance is especially important when patients are not interacting with a therapist or with external devices (e.g., computer-facilitated imagery) for 20–60 min while listening to taped instructions right after a relaxation period. The large differences between actual and mental movement durations found for ADL tasks routinely trained during audiotaped MI (Wu et al., 2010) further raises concerns to that effect. Patients with hemorrhagic strokes imagined the tasks 2–3 times faster than when they executed them physically (Wu et al., 2010), suggesting that they had difficulty in representing mentally complex tasks with accuracy (Guillot and Collet, 2005a). These findings, however, are at variance with those from diverse sources that observed some slowing (about 20–40%) of MI during hand pointing (Malouin et al., 2004c; Stinear et al., 2007) and stepping movements (Malouin et al., 2004c, 2012), especially after right hemispheric strokes (Malouin et al., 2004c, 2012; Stinear et al., 2007) or in patients with sensory deficits (Liepert et al., 2012). The large timing discrepancies reported by Wu et al. (2010) are worrisome and warrant the requirement of regular chronometric checks in future studies.

**Table 4:** **Characteristics of MI training studies for mobility and locomotor activities**.

**^Res Prot^/Study**	***N***	**TSS**	**PP**	**Mental practice**				**Mean change scores**					
			**Hrs**	**Hrs**	**Eval**	**✓**	**Tasks**	**Gait speed**	**Limb**	**ABC**	**Berg**	**DGI**	**TUG (s)**
								**cm/s**	**L %**	**0–100**	**0–56**	**0–100**	
^1B^Yoo and Chung, 2006	E	5 months	2.7	2.7	YES	NO	1	NA	15	NA	NA	NA	NA
Standing	E	23 months	2.0	2.0					17				
	E	8 months	1.5	1.5					21				
^1B^Hwang et al., 2010	E:13	24 months	20	6–10	YES	NO	1	7	NA	46	23	17	5
Gait	C:11	23 months	20					2		10	8	1	3
^1B^ Lee et al., 2011	E:13	Chronic	9	5–9	NO	NO	1	16	NA	NA	NA	NA	NA
Gait	C:11		9	No sham				10					
^1B^ Cho et al., 2013	E:15	45 months	9	6	NO	NO	1	14	NA	NA	NA	NA	8.3
Gait	C:13	46 months		No sham				9					1.6
^2^Malouin et al., 2004a	E:12	Chronic	7 rep	35 rep	YES	YES	2	NA	16	NA	NA	NA	NA
Rising-up/sitting													
^2^Malouin et al., 2009	E:5	2.4 years	100 rep	1100 rep	YES	YES	2	NA	18	NA	NA	NA	NA
Rising-up/sitting	C:4	3.5 years	100 rep	0 rep					−6				
	C:3	2.4 years	0 rep	0 rep					6				
^2^ Deutsch et al., 2012	E:1	10 years	1	5	YES	YES		35		11	NA	NA	2
Gait													
^3^Dunsky et al., 2008	E:17	9–108 months	0	3–4.5	NO	YES	1	15 (8–38)	NA	NA	NA	NA	NA
Gait													
^3^Guttman et al., 2012	E:13	7–55 months	?	2.8–4	YES	NO	1	NA	0	NA	NA	NA	NA
STS													
^2^**Tamir et al., 2007**	**E:11**	**Stage 1.5–3 Hoen and Yahr's stage**	**12**	**12**	**NO**	**NO**	**3**	**NA**	**NA**	**NA**	**NA**	**NA**	**2.5**
**Mobillity**													
**Parkinson D**	**C:10**		**24**										**−0.5**
**^2^Braun et al., 2011**	**E:25**	^*^1.5–3 and <3 Hoen and Yahr's stage	**5**	**6**	**NO**	**NO**	**?**	**3 and 20**	**NA**	**NA**	**NA**	**NA**	**1.5 and 1.3**
**Gait**													
**Parkinson D**	**C:22**		**5**					**14 and 18**					**3.4 and 1.0**

PP, physical practice; TSS, mean time since stroke; Hrs, hours; Eval, MI assessment; ✓, manipulation checks; Tasks, number of tasks rehearsed mentally;

Res Prot 1A, 1B, 2, 3 , research protocols described in Table 1; NC, non-chronic strokes; Limb L, percent of limb loading on the affected side; STS, sit-to-stand; Berg, Berg balance scale; DGI, dynamic gait index; ABC, Activities-specific balance confidence scale; TUG, Timed Up and Go test; NA, not applicable; No sham, no contact time control; rep, repetitions;

* results from 2 analyses: patients in stages 1.5–3 and patients in stages 1.5–2; bold, studies in persons with Parkinson's disease; ?, unspecified.

Although standardized audiotape delivery makes such manipulation checks more difficult, it can be done. Nilsen et al. (2012) conducted manipulation checks on the perspective used during mental rehearsal retrospectively, right after the end of the session. Asking patients periodically what they see or feel is also indicated to check whether the instructions are well-understood (Malouin et al., 2009; Deutsch et al., 2012). Since the enhancing effect of MI on cortical excitability and recruitment patterns depend on imagery quality (Lebon et al., 2012; van der Meulen et al., 2012), such debriefings to control the quality of imagery are particularly important. Their frequency can be reduced with time, as patients get more confident and experienced with MI. Dunsky et al. (2008) used chronometry of imagined walking to gauge engagement during imagery training; likewise chronometry was used during the MI training of rising from a chair and sitting down (Malouin et al., 2004a,b, 2009). It is thus recommended to plan for such MI manipulation checks, especially in older persons who have difficulty concentrating for long periods of time or with persons with impaired cognitive skills who can quickly lose track of ongoing tasks. To conclude, because imagery cannot be directly observed, manipulation checks should be mandatory to ascertain that patients imagine what they are instructed to imagine. Poor adherence could explain the moderate effects of MI training reported in a recent meta-analysis (Barclay-Goddard et al., 2011). The development of guidelines for optimal MI training starts with the control of factors such as MI compliance critical to the interpretation of the results.

### The content and the amount (dose) of MI intervention

**Figure 3:**
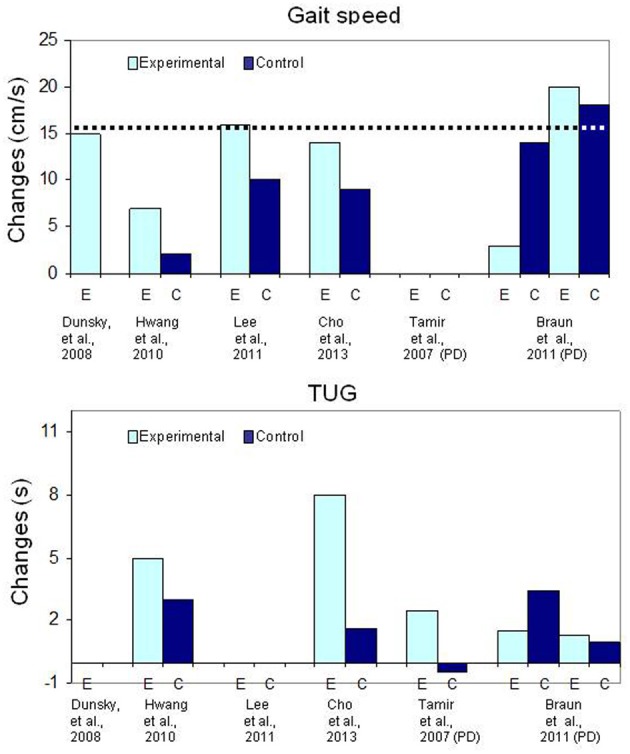
**Bar graph illustrating the mean changes (cm/s) in gait speed (upper graph) and the mean changes (in seconds) for the Timed Up and Go (TUG) test in patients with chronic stroke**. The dotted line (upper graph) indicates the Minimal Clinically Important Difference.

A frequent question about MP in neurological rehabilitation is how much practice (mental and physical) is necessary to promote learning effects? In this section, we try to relate MI training parameters to findings from studies using similar outcomes measures so as to derive indicators for success. In the majority of the studies with a separate mode of MI delivery for training ADL tasks, the same tasks were practiced both physically and mentally and the mental rehearsal part was delivered through audiotaped scripts (mode 1A) preceded by relaxation exercises (studies identified with 1A, first column in Table 3). In fact, spectacular effects (see Figure 2: change scores) were obtained with even less than 2 h of MI training, which corresponds to about 5 or 8 min of mental rehearsal per session (Page et al., 2001; Nilsen et al., 2012). Note also that with 4 h of MI training (Page et al., 2005, 2007) the outcomes were not better compared to those with 2 h (Page et al., 2001). However, for physical practice, the largest gains in ARAT and FMA scores were observed in studies with 18 and 15 h (Figure 2: Page et al., 2001, 2009) as opposed to 6 h (Page et al., 2005, 2007). The effect of more physical practice was also apparent even for patients in the control group when Constraint Induced Therapy (CIT) was provided to all (Page et al., 2009). Thus, the amount of physical practice appears to be determinant in the size of the effects observed when MP and PP are combined in separate sessions. In fact, based on the findings of the only study that examined the effects of different durations of MI training sessions, increasing the duration does not promote better outcomes (Page et al., 2011). This is not surprising since data in athletes suggest the optimal duration to be about 20 min and that a longer session may degrade motivation and increase negative effects such as boredom (Driskell et al., 1994). Likewise, beneficial effects of MI training (less bradykinesia) in patients with Parkinson's disease (Figure 3: lower graph) were obtained only for tasks practiced both mentally and physically (Tamir et al., 2007), further underlining the key role of PP when combined with MP. Thus, the addition of MI training to PP promotes motor performance of upper limb ADL and this performance is further enhanced with more physical practice, but not with more MI training.

These observations are in line with findings that when MI training was provided alone with usual therapy in patient in the subacute phase post-stroke, likely restricting the amount of PP of the tasks rehearsed mentally, it did not yield better outcomes than usual therapy despite very elaborate and intensive MI training (Ietswaart et al., 2011; Timmermans et al., 2013). In fact, when only one task was trained during MI training either with usual therapy (Müller et al., 2007) or when a large number of MP repetitions (mode 2) were combined with a small number of PP (Crosbie et al., 2004), significant gains were reported even at an early stage of recovery post-stroke. A possible explanation could be that when only one task is trained the higher intensity of MI training promotes better learning effects irrespective of the stage of motor recovery.

For locomotor training, however, the addition of physical gait training does not seem to have such an impact on the magnitude of the outcomes (Table 4 and Figure 3). For instance, a mean increase of 15 cm/s (range: 8–38 cm/s) was measured in patients after MI training of gait alone (Dunsky et al., 2008) and most of the gains in gait speed were retained at follow-up, 3 weeks after the end of training. On the other hand, despite 9 h of treadmill training (Lee et al., 2011; Cho et al., 2013), gait speed gains in the MI groups (14 cm/s and 16 cm/s, respectively) were similar to those reported by Dunsky et al. (2008) with MI training alone. In another controlled study, despite 20 h of physical training (unspecified exercises), smaller gains of 7 cm/s and 2 cm/s, corresponding to about half those reported in other studies were found in the MI and control groups, respectively (Hwang et al., 2010). Although the MI training group had gains that were statistically significantly larger compared to the control group, these changes in gait speed, were close to the standard error of the measure, which is 5 cm/s post-stroke (Perera et al., 2006). In addition, the 2 cm/s change in gait speed in the control group shows that physical training alone had no training effect, a rather surprising finding, especially in relatively young subjects (mean age: 46 and 48 years). A possible explanation for the small changes in gait speed after physical training could be related to the very intensive training regimen of 1 h of regular physical therapy and 30 min of MI training daily, 5 days a week for 4 weeks. Negative effects due to overtraining in inactive chronic patients might be responsible for this poor outcome (Sullivan et al., 2007). However, an important finding is that the small changes in gait speed in the MI training group were, however, associated with very large and clinically significant increases in secondary outcome measures such as balance (Berg scale), self-confidence (ABC scale), the Dynamic Gait Index (DGI) and Timed Up and Go (TUG) performance (e.g., Hwang et al., Table 4). The latter observations are surprising and lead one to question why these significant changes were not associated with larger increases in gait speed.

Thus, how much or what type of physical practice is needed? In studies without treadmill training, the effects of some physical practice cannot be discarded totally because the patients were ambulatory and thus continued to walk daily (Dunsky et al., 2006, 2008) and likely increased their walking activities (Cupal and Brewer, 2001; Page et al., 2005). Also, since these studies did not include a control group and that the amount of physical walking outside therapy sessions was not monitored, it is difficult to estimate the role of physical training in the reported gains. Nevertheless, the amount of physical gait practice was likely less compared to the intensive treadmill walking provided elsewhere (Lee et al., 2011; Cho et al., 2013). To conclude, for locomotor MI training, the addition of intensive specific treadmill training did not result in larger gains in gait speed, suggesting that in ambulatory patients, additional physical practice of locomotion is not essential.

**Figure 4:**
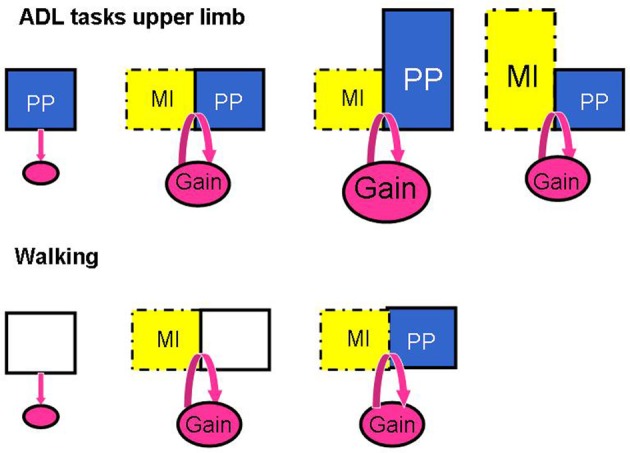
**Schema illustrating the patterns of responses when manipulating the amount of MI training and physical practice (PP) in the studies investigating the effects of MI interventions on ADL tasks of the upper limb and on walking**. The addition of MI to PP promotes motor performance in ADL for the upper limb (Gain) and this performance is further enhanced with more PP, but not with longer MI sessions; for walking, however, MI alone promotes walking speed as much as MI plus treadmill walking.

The above analysis suggests that the interactions between MP and PP are not the same for upper limb and locomotor tasks. These interactions are schematized in Figure 4. The fact that more physical practice may be needed for upper limb ADL tasks is perhaps related to the greater level of motor skill associated with the control of upper limb movements compared to locomotor control which is a rhythmic and automatic activity that is also assisted by the sound leg in its expression.

Thus, it is very difficult to propose an ideal dose for MI training as positive results have been obtained with a variety of regimens. How many repetitions are required to obtain significant gains? Unfortunately, this is a question that has been overlooked so far in most studies examining the effects of MI in disabled populations. Moreover, because training intensity has been mostly reported in hours, we have little information to justify a recommendation for an optimal number of movement repetitions to provide clinically significant gains. We can, however, speculate that it is close to the number found to be successful in healthy persons who learned a new task after hundreds (about 1500) of mental repetitions (Jackson et al., 2003), or in persons with stroke who showed learning effects after about 1100 mental repetitions combined with 100 physical repetitions, for a ratio of 10MP:1PP (Malouin et al., 2009). Also, although no study has compared different ratios of MP: PP, learning effects have been reported with a variety of ratios: 10MP:1PP (Crosbie et al., 2004; Malouin et al., 2009); 3MP: 3PP (Tamir et al., 2007) or 5MP: 1PP (Deutsch et al., 2012). While in theory the more practice, the better, much like excessive physical practice can lead to muscular fatigue, too much mental practice could contribute to mental fatigue. This underlines the importance of monitoring both physical and mental fatigue in rehabilitation. More studies examining specifically dose-related effects of MP and PP will be necessary to gain a better understanding of these factors on motor re-learning following stroke. The gathering of such information is a key for future development of sound clinical guidelines.

### Relaxation component in MI training

Another factor that needs to be explored is the role of the relaxation component often included prior to MI rehearsal, particularly (but not only) in studies with audiotaped scripts (Page, 2000; Page et al., 2001, 2005, 2007, 2009, 2011; Yoo et al., 2001; Dunsky et al., 2008; Deutsch et al., 2012; Nilsen et al., 2012). What are the effects of adding relaxation on MI outcomes? During relaxation, patients are asked to imagine themselves in a warm and relaxing place (beach; bath) and to contract and relax their muscles (progressive relaxation) and in some cases they are asked to stay relaxed until the end of the session (Dunsky et al., 2008). In their study, Yoo et al. (2001) even used EMG recordings to confirm muscle relaxation. While relaxation is less applicable when physical repetitions alternate between series of mental repetitions, it has been almost automatically implemented with other modes of MI training to help the patients perform motor imagery. However, this notion has been challenged by many who state that relaxation is not essential and could even limit imagery-related benefits when used for improving motor learning and performance (Gray et al., 1984; Rushall and Lippman, 1998; Holmes and Collins, 2001). Some authors suggest that relaxation prior to MI training could be used as a starting point, but that it should not be maintained during the entire rehearsal session (Janssen and Sheikh, 1994). Relaxation may be indicated in stressed patients with difficulty imagining or those with poor concentration. Results from a recent study in healthy adults, however, revealed that imagery vividness did not differ in relaxed and aroused conditions (Louis et al., 2011). Moreover, when MI training was carried out in relaxed conditions, it seemed to alter the timing of MI, resulting in longer imagination than execution times (Louis et al., 2011). Some evidence suggests that the level of arousal should be close to that of the real performance (Holmes and Collins, 2001; Guillot and Collet, 2008). Furthermore, beneficial effects on motor performance and skill learning have been found with a novel approach combining real movement with MI, termed dynamic MI (Guillot et al., 2013), which is not compatible with any form of relaxation prior to MI.

In usual practice, listening to relaxation exercises on a pre-recorded audio media has been considered as a neutral procedure and used in studies post-stroke as a sham intervention to control for contact time (Page et al., 2005, 2007, 2011; Nilsen et al., 2012). However, when relaxation has been used as a sham intervention in persons with Parkinson's disease, beneficial effects (Table 4, Figure 3) on walking performance (gait speed) similar to those observed in the experimental group following MI training have been reported (Braun et al., 2011). In the latter study, relaxation was provided in the same session as physical training. It is difficult to determine, however, how (e.g., reducing rigidity or increasing concentration) relaxation promoted a better motor performance in persons with Parkinson's disease. These results do suggest, however, that a relaxation-oriented MI intervention alone may be a factor to consider. Altogether these observations warrant a closer examination of the role of relaxation in the identification of optimal arousal conditions of future MI training rehabilitation protocols.

### Outcome measures for MI training

The selection of reliable and valid outcome measures is always a great challenge. It must take into consideration not only the reliability of a measure but also its validity and responsiveness. Of the many outcome measures available, the challenge is to choose a measure that is appropriate for the evaluation of the specific task that is targeted in the MI training. For instance, gait speed is recognized to be a very robust outcome for measuring the effects of training on walking (Wade, 1992; Richards et al., 1999). Also, because it is a continuous measure, it is possible to monitor progress over a large range of performance (Richards et al., 1995). Moreover, psychometric characteristics of gait speed are well-known, including its Minimal Clinically Important Difference (MCID: Tilson et al., 2010). The MCID is a useful means of evaluating whether the size of the gain in gait speed after an intervention is clinically meaningful, and it also allows for comparison of the effect across studies. For example, in one study, the statistically significant differences found in gait speed gains between groups suggested a better outcome in the MI group (Hwang et al., 2010). However, although there was a statistically significant difference between groups, the intense training regimen yielded small gait speed gains (7 cm/s) well-below the 16 cm/s MCID value (Tilson et al., 2010), and corresponding to about half the gains reported with other protocols of MI training for gait. On the other hand (Table 4: Hwang et al., 2010), gains in secondary outcome measures such as balance (Berg Balance Scale), movement quality (Dynamic Gait Index), obstacle walking and self-efficacy (ABC) measures were significantly larger in the MI group, signifying a role of MI training on the development of balance, self-efficacy, movement strategy and navigation skills which are important for developing walking competency (Salbach et al., 2004). Thus, secondary outcome measures can be helpful for the identification of collateral effects of MI training and to unveil positive findings despite small gains on selected primary outcomes.

The lack of statistical significance between two interventions, however, does not always mean that there are no training effects. For example, in a study involving persons with Parkinson's disease (Braun et al., 2011), no significant statistical differences were found in gait speed gains between a group trained with relaxation and another with MI, which led the authors to conclude that MI training had no effect. However, a closer inspection of the data (Table 4, Figure 3) reveals that both groups had gains in gait speed above the MCID, indicating that both interventions yielded gains that were clinically significant. This example further underlines the importance first, of selecting well-known outcome measures, and secondly, to examine the clinical relevance of the gains when psychometric properties are available.

Secondary outcomes can also further confirm the absence of MI training effects. This was the case in a study examining the effects of two modes of MI training on the learning of a complex mobility task: going down and getting up from the floor (Schuster et al., 2012a). The findings did not confirm that patients learned better when MI was provided in separate or concurrent sessions with physical training because the time to execute the task (outcome measure) diminished as much in the patients in the control group, who practiced the task physically during testing sessions, as in the two MI intervention groups. In addition, there were no significant changes either in the ABC scale or in the Berg Balance scale, further confirming the lack of MI training effects (Schuster et al., 2012a). Both the low intensity of MI training (less than 100 MI repetitions over a 2-week period) and the psychometric properties of the outcome measure (sensitivity and floor effect) could be responsible for the inconclusive findings.

The choice of an outcome measure such as movement speed is not always a valid or optimal measure and will vary according to the task to be evaluated and the aim of the training. For instance, if MI training is used to teach a new strategy (increase the amount of loading on the affected leg) during mobility tasks such as rising-up from a chair and sitting-down, the best marker of improvement is a gain in the amount of limb loading (vertical forces) on the affected side (Engardt et al., 1993; Cheng et al., 2001; Monger et al., 2002; Malouin et al., 2004a,b, 2009). In the early stage of training no change in the speed of movement is expected because it focuses on learning the motor strategy (Engardt et al., 1993; Carr and Shepherd, 1998), but after several weeks of training, improvement in both motor strategy and movement speed can be expected as the affected leg gets stronger (Engardt et al., 1993; Cheng et al., 2001; Monger et al., 2002). On the other hand, an increase in movement speed without a concomitant improvement in motor strategy signals a compensatory strategy with the sound leg (Engardt, 1994). Therefore, a gain in movement speed without an increase in limb loading on the affected side after MI training of sit-to-stand (Guttman et al., 2012) suggests that the MI training protocol did not promote the learning of the novel motor strategy. Likewise, the use of the knee extensor muscle activity (EMG alone) as an indicator of vertical force distribution between the paretic and non-paretic limbs as a means of assessing an improved motor strategy can be questioned (Oh et al., 2010), and requires prior validation.

The selection of outcome measures becomes even more complex when they are not specific to the tasks trained. For instance, in the RCT studies that evaluated the effects of MI training of several tasks, it is not clear how the ongoing recovery of the patients leading to, for example, increased muscle strength, better antagonist muscle coordination, or inter-segmental limb coordination, relate to the outcome scores from various tests using ordinal scales such as the Arm Research Action test (ARAT), Fugl-Meyer Assessment (FMA) or Motricity Index (MI). Although these clinical scales are very reliable (Lyle, 1981; Duncan et al., 1983; van der Lee et al., 2001; Lang et al., 2008), and provide a global score of performance, it is at times difficult to understand how they relate to specific changes in motor behavior. Examining ARAT subscales may, however, help pinpoint areas of improvement (e.g., pinch, grasp, or reach). Moreover, when impairment outcomes such as the FMA are used concurrently with quantitative measures such as those from the Jebsen test (Jebsen et al., 1969), they confirm a translation of training to motor performance (Müller et al., 2007; Nilsen et al., 2012).

When examining Figure 2, it is difficult to explain the modulation of the scores across studies sharing similar training protocols. For example, in a recent study (Page et al., 2011) MI training induced very small ARAT and FMA changes scores (not clinically meaningful) compared to those in previous studies (Page et al., 2001, 2005, 2007, 2009) despite similar MI training protocols. Another question concerns the relationship between the tasks rehearsed and the outcomes. Weight shifting exercises on the affected arm (Page, 2000) led to as much FMA gains as did tasks such as reaching and grasping, turning a page or writing (Page et al., 2007), further suggesting the non-specificity of such outcomes. Lastly, how can we explain so much variability in the ARAT change scores in control groups across studies (Page et al., 2001, 2005, 2007) and why are these changes so small despite 6–18 h of physical training (Page et al., 2001, 2007)? The small impact of usual therapy on patients in control groups (Riccio et al., 2010; Table 3) raises concerns about both the intensity of therapy (Lang et al., 2009) provided in early rehabilitation and the sensitivity of selected outcome measures (Table 3 and Figure 2).

Few clinical studies have examined the specificity of MP training, by focusing on a single ADL task. The use of quantitative outcome measures such as a computerized test for assessing reaching times, the Box and Block test and the Purdue pegboard test might help gain a better understanding of the specific effects of MI on function. Crajé et al. (2010) showed that MI training of several functional activities could result in specific effects such as improved reaching and grasping but not of fine dexterity. Such findings are of interest because they help explain the specific MI training effects on motor function rather than having a global total score of grasping, reaching and pinching. Again, the psychometric properties of a test are useful to gauge the importance of the change not only statistically but also clinically. For instance, a gain of 7 blocks in persons with stroke on the Box and Block test translates to improvement of daily physical functioning (McEwen, 1995).

It is also important to assess generalization effects of MI on function. In a pilot study (Müller et al., 2007) the intensive MI training of sequential finger movements for 30 min per day, 5 days per week for 4 weeks, led to an increase in the peak torque of the pinch grip that was comparable to that obtained with physical training. Moreover, this increase in strength was generalized to better function of the upper extremity as measured by the timed items in the Jebsen test, (Table 3) that assesses the time taken to execute seven upper extremity tasks (Jebsen et al., 1969). Assessment of other outcomes such as concentration, motivation, or self-efficacy should also be considered because it is also important to evaluate the effects of MI on behavioral and cognitive functions (Hwang et al., 2010; Deutsch et al., 2012). Measuring the effects of MI on movement quality or on limb use with accelerometers (Timmermans et al., 2013) is also of interest because MI has been shown to lead to a spontaneous use of limbs trained with MI (Cupal and Brewer, 2001; Page et al., 2005).

To summarize, there is a need of studies that provide a clear link between MI training and specific parameters of motor function through quantitative and valid outcome measures to enable the development of evidence-based guidelines for MI training. In addition, secondary outcomes examining other components of behavior (motivation, self-efficacy, mood etc.) are useful because they extend our understanding of the mechanisms contributing to the positive effects of MI training.

### Group heterogeneity

In clinical studies, the comparability of patients from the intervention and control groups in a given study or among studies is critical to the interpretation of the results. For example, as illustrated in Table 3 there is a large disparity in ARAT gains across studies. A possible explanation for this is the variability in patient level of activity limitations (baseline ARAT scores). For instance, in the study comparing the effects of Constraint Induced Therapy (CIT) with and without the addition of MI, which induced very large gains (Page et al., 2009), the standard deviation (SD) of the ARAT scores at baseline for both groups was very small (*SD* of 1.1 and 1.4, respectively) indicating that the 5 patients in each group had initially a similar level of activity limitation, and, as reflected by the SD of the mean change scores, they all improved in a similar manner indicating that all were good responders. In contrast, in the studies with larger patient groups at an earlier stage of recovery (Bovend'Eerdt et al., 2010; Ietswaart et al., 2011; Braun et al., 2012; Timmermans et al., 2013), the baseline ARAT scores had a large SD, indicating heterogeneity in the activity limitation level of the groups. Consequently, one can expect a variable response to training. Such variability in training response is well documented in studies providing individual data. For instance, in the Dunsky et al. (2008) study, although the mean gain in gait speed was 15 cm/s, the individual gains ranged from about 10 cm/s to 38 cm/s. The large *SD* of 15.66 for a mean gain of 16 cm/s in gait speed also reflects the large variability in individual responses to the same MI training (Lee et al., 2011). Likewise, in a series of case studies (Crosbie et al., 2004), individual gains extended over a wide range, also indicating that the group consisted of responders and non-responders.

The level of impairment also needs to be taken into consideration in data analysis and interpretation. As an example, in the Braun et al. study (2011) in persons with Parkinson's disease, larger effects of MI training were found in sub-groups of less impaired patients. Thus, additional comparisons between sub-groups of responders and non-responders can help tease out factors associated with the positive outcomes (e.g., sensory or cognitive deficit, level of motor impairment, anxiety, motor imagery ability, etc.). For instance, patients who learned to increase the loading on the affected leg during the rising-up from a chair and sitting down tasks were those with a good short term working memory (Malouin et al., 2004b). Correlative analyses between primary motor outcomes and secondary outcomes, such as anxiety and self-confidence, could serve as indicators on how MI is working (Cupal and Brewer, 2001). With analyses looking only at averaged data, important information about the type of patients most likely to benefit from MI training can be missed.

### The selection of patients

The selection of patients is another factor likely to influence the integration of MI training into clinical practice. The best example comes from a recent study that introduced MI training in the regular rehabilitation of older patients (mean age 78 years) who had suffered a recent stroke and lived in a nursing home (Braun et al., 2012). First, therapists found that teaching MI and assuring compliance of the patients proved difficult in these older and frail persons who needed long instruction periods that often induced frustration (Braun et al., 2010, 2012). In addition, it was not possible to implement the training without increasing therapy time, a downside often not acceptable within clinical settings. Also, older persons without previous exposure to MI appear to be less positive and less open to engage in these demanding and abstract procedures. Implementation of MI for the multitude of tasks practiced in regular therapy has also proven quite difficult with poor compliance by both younger (mean age 50 years) patients with stroke and therapists (Bovend'Eerdt et al., 2010). The poor compliance in these patients was explained in part by practical reasons (e.g., therapists on vacation), but it was also linked to patient-specific issues such as cognitive problems (Bovend'Eerdt et al., 2010). Although age as such is not necessarily a deterrent, the cognitive limitations associated with age and co-morbidities do contribute to poor compliance.

In fact, screening for cognitive problems and MI ability should be mandatory given the role of working memory in MI and documented working memory problems with aging and stroke (Malouin et al., 2004b, 2010, 2012; Schott, 2012). Both reduced working memory and poor attention skills can make the teaching of MI more difficult (Braun et al., 2010, 2012). Screening should also take into account language disorders that hamper the capacity to understand the instructions. It could also be that like other adjunct therapies, MI training may not be suitable or appealing to all patients because it requires first, to believe in the process, and then to accept to make the mental effort to engage in MI, a task that can be too demanding in some cases. Demands are also made on the therapists, who need to acquire some knowledge and understanding of the processes underlying MI training and then to develop expertise in its implementation prior to training patients. Such requirements may not be appealing or suitable to all.

Compliance to treatment requires the ability to imagine and it is surprising that MI ability is so rarely assessed (Tables 3, 4). A possible reason is that because of its covert nature, MI needs to be assessed by different strategies. This has resulted in the development of several sophisticated approaches for assessing MI ability (see Guillot and Collet, 2005b; Heremans et al., 2008; Collet et al., 2011), that are not readily amenable to clinical settings when the therapist needs to decide whether a patient is able to engage in MI. Simpler and clinically amenable tests, however, are available. In our experience, screening can be done within a short time frame with an MI questionnaire and chronometric tests (Malouin et al., 2008a,b; Malouin and Richards, 2013). First, the administration of the KVIQ (Kinesthetic and Visual Imagery Questionnaire), informs on whether the patient is able to generate vivid images of simple movements (Malouin et al., 2007). Although, the questionnaire remains a subjective tool [e.g., such as a Visual Analogue Scale (VAS) for pain], the examiner can test whether the rating provided by the patient for a given item is genuine by asking the patient to provide details about the perspective (e.g., what part of the body is seen) and about the vividness of image (clarity, color etc.) and sensations (of joint, skin, muscles) perceived. Such a debriefing is important initially to make sure that MI instructions and scale ratings are understood correctly. Also, the pattern of responses can provide additional indices. If the patient always gives the same rating or always answers very quickly without concentrating, it suggests that more debriefing is needed. In other words, the administration procedures of the KVIQ, as well as the score provide some information about the ability of a person to engage in MI.

The MI questionnaire KVIQ was developed for testing persons with physical disabilities (Malouin et al., 2007) and it includes items that can be tested in sitting which makes this tool more accessible to persons with sensorimotor disturbances and balance limitations. The reliability and validity of the KVIQ have been documented in patients post stroke (Malouin et al., 2007, 2008b), in persons with Parkinson's disease (Randhawa et al., 2010) and a German version was recently validated (Schuster et al., 2012b). The validity of imagery questionnaires for assessing MI ability has been questioned because of the subjective nature of self-reported ratings (Lotze and Halsband, 2006; Sharma et al., 2006). However, over the last few years, studies examining brain activation patterns (fMRI and EEG) and corticospinal excitability (TMS) have found significant correlations between imagery scores and brain activity (Lorey et al., 2011; Williams et al., 2012; Vuckovic and Osuagwu, 2013). Note also that similar positive correlations have been described in persons with spinal cord injury (Alkadhi et al., 2005) and upper limb amputation (Lotze et al., 2001). However, MI vividness is a single dimension of MI ability and it is recommended from a clinical standpoint to use additional tests such as a chronometric test to further confirm the ability of a patient to engage in MI. For instance, comparisons between the duration of imagined and real movement (mental chronometry) indicate if the patient has a good temporal representation of the tasks being rehearsed mentally (see Malouin et al., 2008a; Malouin and Richards, 2013). Consequently, the first step to improve compliance to MI training should be to examine the MI ability of potential participants. However, because MI ability improves in the first weeks after stroke (de Vries et al., 2011), repeated evaluations are recommended before rejecting potential participants on this basis. As mentioned above, we need to develop criteria to guide the use of MI and the minimal requirement should be that patients be able to engage in MI.

### Mental rehearsal: audiotape scripts versus a guided one to one approach

**A**nother factor that requires attention is the nature of MI instructions that differ greatly across studies. When MI training is carried out with audiotaped instructions, the patient listens to a script describing step by step how a task should be achieved (strategy), as well as the images and sensations during the completion of the task. Also, the wording can be very motivating, as it encourages patients to see the arm and hand moving freely and easily, and that the task is being performed effectively (Nilsen et al., 2012). Moreover, since the scripts are not always constructed to mimic movements in real time, participants are encouraged to repeat the movements at their own speed. For the task of drinking from a cup, participants, after being instructed to focus on reaching the cup, lifting the cup of the table and to bring it to the mouth, are then instructed to take a sip of water, then another sip and another sip and so on until they are feeling refreshed and are done drinking (D. Nilsen personal communication). This means that the number of repetitions can be quite variable from one patient to another and from day to day and suggests that the training strategy is not about reaching a certain number of repetitions. It seems rather, that patients gain some self-confidence in how the task should be performed (problem solving) and can be done successfully (motivation, reward), paving the way for the next physical practice session and suggesting that priming effects of MI take place *implicitly* during physical practice.

In contrast, in one to one guided MI, less emphasis is put on the emotional aspect, instead, the instructions are adapted to individual needs and limitations and more details *(explicit)* on how the movement should be performed are given. This is a very dynamic approach and requires a close interaction between the patient and the therapist who guides the patient throughout the stages of motor learning during both mental and/or physical rehearsals (Rushall and Lippman, 1998; Malouin et al., 2004a,b, 2009; Ietswaart et al., 2011; Deutsch et al., 2012; Timmermans et al., 2013). This mode is quite demanding for therapists who need time to introduce patients to MI training, a good knowledge about MI processes and prior familiarization with MI. Technical support is also used at times to illustrate what should be imagined. Mirror therapy (Ietswaart et al., 2011) or watching videos to learn about walking, or to identify their own gait problems from videos taken at different intervals (Hwang et al., 2010; Lee et al., 2011) and interacting with a computer and hardware devices for visual feedback of limb loading during a familiarization period in the first training session (Malouin et al., 2004a,b, 2009; Oh et al., 2010) are examples. It can involve actively imagining with the use of mirrors (Stevens and Stoykov, 2003; Ietswaart et al., 2011) or computer-facilitated imagery (Stevens and Stoykov, 2003). In addition, when mental and physical repetitions are combined within the same session, regular feedback about the physical performance is given by the therapist who also makes imagery checks with chronometry or some debriefing about the imagery to control for imagery quality (Crosbie et al., 2004; Malouin et al., 2004a,b, 2009; Deutsch et al., 2012). Thus, this approach likely puts a heavier demand on participant concentration and attention skills compared to audiotape delivery preceded by relaxation.

## Integrating MI training in current practice: a framework

Although motor learning theories and neurological mechanisms are outside the scope of this review, one can speculate about how MI training can improve motor performance. Page et al. (2005) proposed that the motor improvement observed after MI training with audiotape scripts resulted from an increase in spontaneous motor activities. They found that patients after MI training used their affected limb more often suggesting that part of the gains observed could be attributed to the additional physical practice. Likewise, athletes after a rehabilitation program with MP for knee injuries demonstrated greater motivation to engage in physical therapy, which may have led to better rehabilitation outcomes. In the latter case, the subjects in the MP group had not only greater knee strength but this gain in strength was also associated with less re-injury anxiety than those in the control group, indicating that the MI training had effects on both psychological and physical rehabilitation outcomes and that reduction in re-injury anxiety and pain enabled the participants to relax and engage more fully in rehabilitation.

As demonstrated in animal models (e.g., Nudo et al., 1996) and in humans (Pascual-Leone et al., 1995; Lafleur et al., 2002; Jackson et al., 2003) the rehearsal of motor actions through physical and mental practice can induce brain changes (plasticity) associated with skill learning. As nicely demonstrated by Pascual-Leone et al. (1995), the changes in cortical sensorimotor maps after mental training are similar to those obtained with physical training. Since mental training has preparatory effects and increases the efficiency of subsequent physical training (Pascual-Leone et al., 1995) their combination is expected to yield best results. Although the optimal MI training approach remains unclear at this time, and that beneficial effects of MP on motor performance have been reported with all three modes of MI delivery, instead of using one exclusively, it might be reasonable to examine whether they could complement each other when used sequentially along the rehabilitation process.

**Figure 5:**
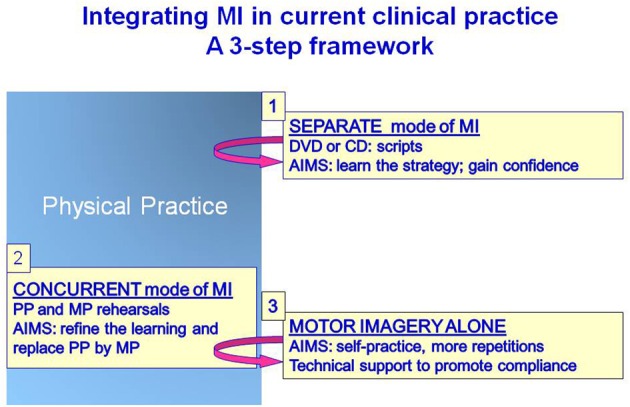
**Schematic representation of a framework for integrating MI training in current clinical practice**.

Is the integration of MI training in rehabilitation programs a mission impossible? Based on previous studies, several features can hamper this integration: an early stage of motor recovery (e.g., implying spontaneous recovery), a wide spectrum of tasks, cognitive limitations, compliance with MI training, and relatively inexperienced therapists in the use of MP. Therefore, a first action would be to control factors that can be controlled such as: screening for impeding cognitive problems, assessing MI ability, determining an optimal number of tasks, training the therapists, planning for manipulation checks of MI and identifying valid primary and secondary outcome measures, while taking into account the advantages of each mode of MI administration along the rehabilitation continuum. The following strategy proposes a 3-step framework for the integration of MI intervention in current clinical practice (Figure 5).

### Step 1: introduction to MI training

Adding mental exercises to a training session that can sometimes be considered already too short can be viewed as too daring, especially if the patient is still a little confused and fatigues rapidly. So, at this stage, MI training should not be too demanding, both in terms of time and mental effort to learn the procedures. Therefore, to avoid removing time dedicated to regular therapy sessions, one might consider adding MI in a separate mode of administration with audio scripts (CD, MP3, etc.), audiovisual support, or web or stand-alone computer applications. This mode requires less professional resources once the scripts and the material are developed. At this stage, the aims would be to introduce MI, to familiarize the patient with MI, and to apply MI training to one or two tasks with scripts to learn the movement strategy and gain confidence for successful performance. The role of this MI training would be to prepare for the next rehabilitation training session when the same task is practiced physically to promote learning. This part of MI training would be the equivalent of performance preparation described in athletes prior to competition, and likewise the focus of training should be put on factors that enhance performance such as strategy, motivation and concentration (Paivio, 1985; Rushall and Lippman, 1998; Munzert and Lorey, 2013).

### Step 2: insertion of MI combined with PP in current training sessions

Once the patient is well-familiarized with MI, the next step could then be to gradually introduce mental rehearsals of tasks that are also trained physically in regular training sessions (starting with a simple task and then increasing the number and complexity). This part relates to skill learning and the idea is to increase the number of repetitions through MI. It requires a close interaction with the therapist giving instructions adapted to individual needs and limitations (i.e., a more explicit approach). With the concurrent mode of MI intervention, feedback about physical performance is given by the therapist who also makes regular imagery checks to control for compliance and quality of MI. Because this approach is more demanding at the beginning, it is advised to start with a small number of mental repetitions and the ratio between mental and physical repetitions should be gauged according to individual capacities. This procedure has been integrated successfully into regular practice (Crosbie et al., 2004; Tamir et al., 2007). In one study, better outcomes were found in the persons with Parkinson's disease who had replaced half of the physical repetitions by mental repetitions (ratio of 3MP:3PP) indicating that the MI training group had larger gains despite less physical repetitions. The MI training targeted three tasks without increasing therapy session time. Furthermore, this approach proved particularly successful for the more physically demanding mobility tasks (Tamir et al., 2007). Likewise, at a ratio of 10MP to 1PP, training of reaching and grasping has also been successfully introduced into regular therapy without additional treatment time (Crosbie et al., 2004). At this stage the idea is to use mental rehearsal to promote the next physical execution of the task and the physical rehearsal provides sensory feedback to promote the vividness of the task rehearsed mentally.

### Step 3: self-practice for increasing the number of repetitions

There is certainly a need to demystify MI training and to test its use in daily practice, but in small dose that focuses on a few tasks in a well selected patient population. However, since the basic ingredient of motor learning is the high number of repetitions (Pascual-Leone et al., 1995; Jackson et al., 2003; Allami et al., 2008; Reiser et al., 2011), we have to find ways of increasing the mental repetitions in a stimulating fashion outside formal therapy sessions. Homework are not very appealing to most and for those who try, it does not last very long (poor adherence). Thus, we need to develop dynamic interactive applications easy to use anywhere (i.e., electronic tablets) of computer-facilitated imagery (Stevens and Stoykov, 2003) to guide the patients through mental rehearsal routines of different levels of difficulty. This progression should include manipulation checks to control for imagery quality and compliance throughout each routine. This step is critical for developing some autonomy so that mental practice can be continued at home (Jackson et al., 2004).

## Future research targets

In the future, more effort should be put into clarifying the specific effects of MI training with each mode of MI delivery to determine their respective advantages and also to identify the characteristics of patients most likely to benefit from each type of delivery. We need to understand the role of factors such as the content and amount of training (physical and mental), relaxation, instructions and valid outcome measures (motor and behavioral). Motor learning theories in relation with the modes of MI delivery should also be examined. Because of the functional similarities between MI and motor execution, one would think that they share similar rules relative to motor learning, but recent findings in healthy adults have shown that while task variability promotes skill learning with physical practice, it does not have the same effect with mental practice (Coelho et al., 2012).

Much can be learned from the work accomplished to date, but prior to initiating large multicenter RCTs, so demanding both financially and in human resources, well-designed and hypothesis-driven pilot studies are also needed to clarify the impact of the many factors that influence MI training outcomes. For example, in one study with 14 patients, tested on 4 consecutive days, it was possible to compare the effects of four imagery protocols for MI training of walking (Kim et al., 2011). The findings of such studies contribute to the refining of future experimental paradigms in RCTs (Dobkin, 2009). For patients with difficulty in engaging in MI, the enhancing effects of brain stimulation, such as Transcranial Direct Current Stimulation (Ang et al., 2012; Foerster et al., 2013) or peripheral stimulation (Saito et al., 2013), on MI may prove to be useful. We also need to examine more closely the brain changes associated with MI training and also to characterize the effects of different modes of MI delivery using neuroimaging methodology such as Near Infrared Spectroscopy (NIRS) that is more amenable to recording changes in brain function during functional activities (Mihara et al., 2013).

## Conclusion

This review delved into the details of research protocols using MI and uncovered several issues that should be addressed in future studies. The following points are important for future comparisons between studies, but also to facilitate the transfer of experimental findings to clinical settings. A better understanding is needed of factors contributing to training effects in relation to each mode of MI delivery, as is clarification of their respective impact at various stages (subacute-chronic) of motor recovery to guide their use. Thus, it is important to systematically record the content and quantity of training regimens (physical and mental) to gain some understanding of the dose-related responses associated with each mode of MI delivery. Also, it is essential to select patients that can engage in MI and to use manipulation checks to confirm their adherence to MI training. The selection of valid outcome measures specific to the trained tasks is a central issue; the choice of outcomes should be based on psychometric properties such as reliability and sensitivity to ensure detection of clinically significant changes. Finally, sub-group analyses are required to characterize responders from non-responders to a given MI training. This review proposes a framework to assist in the integration of MI into rehabilitation programs. We know little about the potential use of MP in persons with subacute stroke. Thus, the challenge has now shifted towards the demonstration that MI training can enhance the effects of regular therapy in persons with subacute stroke during the period of spontaneous recovery. The proposed framework is only a starting point. The combined effort of clinicians and researchers is essential to put it to the test, and to adjust it in accordance with ongoing clinical findings.

### Conflict of interest statement

The authors declare that the research was conducted in the absence of any commercial or financial relationships that could be construed as a potential conflict of interest.
